# Tailoring Hydrogenation to Enhance Defect Suppression and Charge Transport in Hydrogenated Amorphous Silicon for Flexible Photodetectors

**DOI:** 10.1002/advs.202504199

**Published:** 2025-06-23

**Authors:** Ye‐ji Jeong, Kyeong‐jin Hyun, Hee‐Won Jang, Jong‐won Yun, Yong‐Hun Kim, Woon Ik Park, Soo‐Won Choi, Jung‐Dae Kwon

**Affiliations:** ^1^ Energy & Environment Materials Division Korea Institute of Materials Science Changwon Gyeongnam 51508 Republic of Korea; ^2^ Department of Materials Science and Engineering Pukyong National University Busan 48513 Republic of Korea; ^3^ Department of Materials Science and Engineering Pusan National University Busan 46241 Republic of Korea

**Keywords:** defect passivation, flexible electronics, hydrogenated amorphous silicon, low temperatures, photodetectors

## Abstract

Visible‐light photodetectors (VPDs) garner significant attention due to their diverse applications in optical communication. However, conventional VPDs struggle to achieve both transparency and flexibility, limiting their use in emerging technologies. Hydrogenated amorphous silicon (a‐Si:H) offers a promising platform for flexible optoelectronics for compatibility with substrates, although temperature reduction causes degradation of electrical and optical properties due to insufficient hydrogen passivation. In this study, the effect of the hydrogen‐to‐silane (H_2_/SiH_4_; *f* ratio) gas is systematically investigated ratio on the microstructural, optical, and electrical properties of a‐Si:H films synthesized at an ultra‐low temperature of 90 °C using plasma‐enhanced chemical vapor deposition (PECVD). Raman and Fourier‐transform infrared (FT‐IR) spectroscopy reveal that an optimized H_2_/SiH_4_ ratio minimizes Si─H_2_ bonding, effectively reducing defect density and improving film stability. Spectroscopic ellipsometry confirms that this ratio optimizes the refractive index and optical bandgap, enhancing light absorption. Electrical measurements demonstrate that photodiodes with the optimized a‐Si:H layer exhibit superior photosensitivity and suppressed dark current (*f*_2_: 20.6 and *f*_8_: 2.70 × 10^−10^ A, respectively), attributed to improved carrier transport and reduced Shockley–Read–Hall (SRH) recombination. Furthermore, flexible photodetectors maintain high mechanical reliability under repeated bending cycles. These findings highlight the potential of ultra‐low‐temperature PECVD a‐Si:H films for high‐performance, flexible photodetectors.

## Graphical abstract

Hydregen content was tailored during ultra‐low‐temperature plasma‐enhanced chemical vaper deposition (PECVD) to deposit hydrogenated amorphous silicon (a ‐ Si:H) films, and the effect of the hydrogen dilution ratio on defect suppression was analyzed. The optimized condition yielded a photodetector with a high responsivitiy of 338 mA/W and excellent mechanical stability, enabling a flexible visible‐light photodetector (VPD).

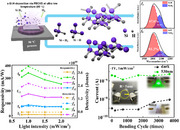

## 1 Introduction

Visible‐light photodetectors (VPDs), which convert optical signals into electrical responses, have gained significant attention in various applications, including optical communication, medical diagnostics, environmental monitoring, autonomous vehicles, and image sensors.^[^ ^1^ ^]^ Conventional VPDs are primarily fabricated using inorganic materials, such as Si, Ge, and compound semiconductors.^[^ ^2^ ^]^ However, these materials face several challenges, including high processing temperatures, complex fabrication processes, and high cost.^[^ ^3^ ^]^ Furthermore, they are intrinsically unsuitable for the development of transparent and flexible devices, necessitating the exploration of alternative materials for such applications.^[^ ^4^ ^]^ Among promising alternatives, hydrogenated amorphous silicon (a‐Si:H), organic materials, and metal halide perovskites have been extensively studied.^[^ ^5^, ^6^, ^7^ ^]^ In particular, a‐Si:H offers several advantages, including chemical stability, nontoxicity, low production cost, scalability, and compatibility with Complementary Metal‐Oxide‐Semiconductor processes.^[^ ^8^ ^]^ Moreover, a‐Si:H‐based VPDs typically adopt a p‐i‐n diode architecture, which improves the response time compared with p‐n junctions and avalanche diodes while mitigating the influence of carrier diffusion through an expanded depletion region.^[^ ^9^ ^]^

a‐Si:H is conventionally deposited via plasma‐enhanced chemical vapor deposition (PECVD) at temperatures exceeding 200 °C.^[^ ^10^ ^]^ However, most flexible substrates, such as polydimethylsiloxane (PDMS), polyethylene terephthalate, and polyethylene naphthalate (PEN), exhibit glass transition temperatures below 125 °C, thus requiring the deposition of a‐Si:H at temperatures below 125 °C for flexible VPDs.^[^ ^11^, ^12^ ^]^ Under low‐temperature PECVD conditions, the electrical and optoelectronic properties of a‐Si:H films can be compromised owing to the degradation of film quality.^[^ ^13^ ^]^ This degradation arises from insufficient hydrogen passivation, which introduces irregularities into the a‐Si:H matrix and results in structural defects.^[^ ^14^ ^]^ These defects lead to increased thermal losses via Shockley–Read–Hall (SRH) recombination, reduced carrier mobility and photodetector sensitivity, and elevated dark currents within the intrinsic layer, collectively deteriorating the photodiode performance.^[^ ^15^, ^16^, ^17^ ^]^ Therefore, optimization of hydrogen passivation and film thickness is crucial for the development of highly efficient, flexible VPDs under low‐temperature conditions.

In this study, we developed a‐Si:H‐based flexible VPDs fabricated via an ultra‐low‐temperature process at 90 °C. A key innovation in flexible VPDs lies in the application of a photoresist (PR) as a sacrificial layer, enabling precise device pattern definition through a straightforward acetone‐based lift‐off technique. The undesired portions of the film were removed along with the PR, which prevented thermal degradation or deformation of the PR during high‐temperature deposition. This eliminated the need for additional dry or wet etching processes, thereby simplifying the fabrication and enhancing the compatibility with flexible or thermally sensitive substrates. To advance VPD performance, we systematically explored the properties of intrinsic a‐Si:H (i‐a‐Si:H) films prepared at an extremely low temperature of 90 °C by varying the hydrogen‐to‐silane gas flow ratio during deposition. The effect of the hydrogen content in i‐a‐Si:H was investigated by structural, optical, and electrical analyses. The optimized p‐i‐n VPD demonstrated a responsivity of 338 mA W^−1^, rivaling that of its high‐temperature‐processed counterparts grown on rigid substrates as a previously reported study,^[^ ^18^ ^]^ showing comparable excellence and reliability. Moreover, flexible a‐Si:H‐based VPDs exhibited exceptional mechanical reliability in repeated bending cycle tests with a bending radius of 5 mm. These findings highlight the successful realization of flexible p‐i‐n diodes with excellent optical performance under ultralow deposition temperature conditions, paving the way for advanced flexible photodetector applications.

## 2 Experimental Section

### 2.1 Fabrication of a‐Si:H Based Flexible VPDs

**Figure 1:**
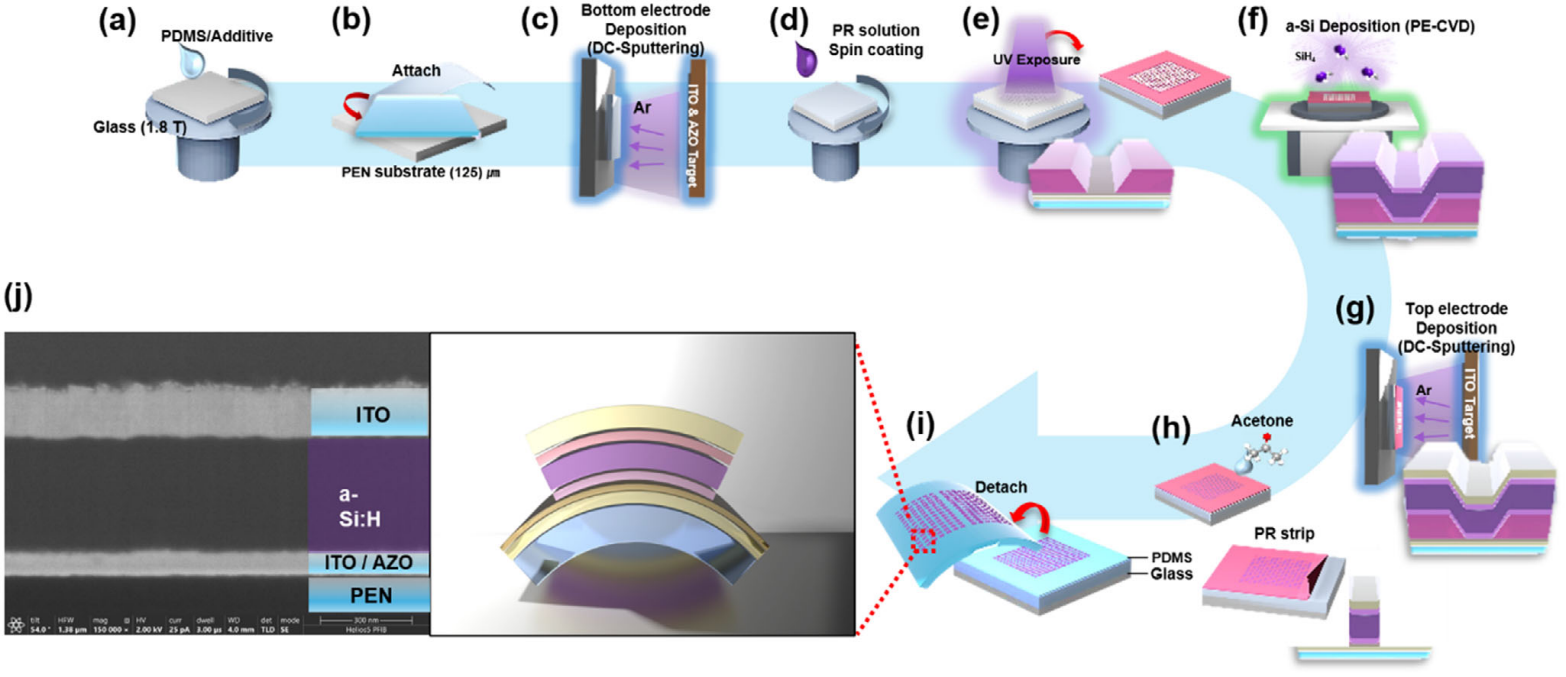
Schematic illustration of Visible light photodetectors (VPDs) fabrication: a) spin‐coating process of polydimethylsiloxane (PDMS) sticker onto glass, b) attaching the polyethylene naphthalate (PEN) film onto PDMS sticker, c) sputtering deposition of bottom indium tin oxide (ITO)/ aluminum‐doped zinc oxide (AZO) electrode, d) spin‐coating process of photoresist (PR), e) UV‐exposure for photolithography, f) deposition of a‐Si:H layers using plasma‐enhanced chemical vapor deposition (PECVD), g) deposition of top ITO electrode, h) acetone‐based lift‐off method with PR strip, i) detaching the flexible VPDs from glass substrate, and j) schematic structure and SEM image of flexible VPDs.

**Figure** **1** summarizes the experimental procedure for the fabrication of the flexible a‐Si:H‐based VPDs. Initially, soda‐lime glass substrates (50 × 50 mm^2^, thickness 1.8 mm) were spin‐coated with PDMS (Figure 1a). To fabricate flexible devices, a 125‐µm‐thick PEN was adhered to the glass substrate (Figure 1b). The bottom electrode, consisting of indium tin oxide (ITO; 100 nm) and aluminum‐doped zinc oxide (AZO; 10 nm), was deposited via direct current magnetron sputtering at low pressure (2.0 × 10^−4^ Pa) and room temperature (25 °C) (Figure 1c). The positive PR was spin‐coated onto the bottom electrode, exposed to UV, and then developed to form the desired pattern (Figure 1d,e). The desired pattern (active area) of the photodetector was precisely defined as 900 µm × 1500 µm using photolithography to achieve uniform device dimensions and reproducible performance evaluation. Subsequently, p‐type hydrogenated amorphous silicon carbide (p‐a‐SiCx:H; 15 nm), i‐a‐Si:H (400 nm), and n‐type hydrogenated amorphous silicon oxide (n‐a‐SiOx:H; 30 nm) layers were sequentially deposited using PECVD at extremely low temperature of 90 °C on the patterned substrate (Figure 1f). The p‐ and n‐layers were deposited using a radio frequency of 13.56 MHz, whereas the i‐layer was deposited at a considerably high frequency of 40.68 MHz. SiH_4_ and H_2_ gases were used as precursors, with the doping gases B_2_H_6_ and PH_3_. CO_2_ was used for the n‐type layer, and CH_4_ was used for the p‐type layer. The detailed deposition conditions and parameters are presented in **Tables** **1** and **2**. The top electrode, consisting of ITO (200 nm), was deposited via sputtering (Figure 1g). The active area (0.0135 cm^2^) was determined using an acetone‐based PR stripping process (Figure 1h). Finally, the diodes deposited on the PEN substrate were delaminated from the glass, resulting in flexible p–i–n diodes (Figure 1i).

**Table 1:** Deposition condition of gas flow ratio for a‐Si:H in PECVD process.

	H_2_/SiH_4_	CO_2_/SiH_4_	B_2_H_6_/SiH_4_	PH_3_/SiH_4_	CH_4_/SiH_4_
p‐a‐SiCx:H	15	−	0.9	−	0.6
i‐a‐Si:H	2, 4, 8, 12	−	−	−	−
n‐a‐SiOx:H	160	0.6	−	1	−

**Table 2:** Detailed deposition parameter of a‐Si:H films via PECVD process.

Material	Deposition Rates [Å/s]	Working Pressure [Pa]	Plasma Power [W]	RF Power Density [mW cm^−2^]
p‐a‐SiC_x_:H	0.28	79.99	50 (RF)	105.21
i‐a‐Si:H (*f*_2_)	1.38	53.33	20 (VHF)	42.08
i‐a‐Si:H (*f*_4_)	1.02	53.33	20 (VHF)	42.08
i‐a‐Si:H (*f*_8_)	1.10	53.33	20 (VHF)	42.08
i‐a‐Si:H (*f*_12_)	1.07	53.33	20 (VHF)	42.08
n‐a‐SiOx:H	1.55	239.98	50 (RF)	105.21

### 2.2 Characterization

To evaluate the properties of the i‐layer, 400‐nm‐thick i‐a‐Si:H films were prepared on glass substrates at various H_2_/SiH_4_ gas flow ratios. The film thickness was measured by spectroscopic ellipsometry (SE MG‐1000, NanoView), and the microstructure was analyzed via Raman spectroscopy (JP NRS‐3300, JASCO). The bonding configurations of the i‐a‐Si:H films were investigated using Fourier‐transform infrared (FT‐IR) spectroscopy (Nicolet iS 10, Thermo Fisher Scientific Inc.). The optical bandgap (E_g_) was determined using Tauc's method,^[^ ^19^, ^20^ ^]^ and the transmittance of the i‐a‐Si:H films was analyzed using a UV–vis spectrometer (Cary 5000, Varian). Dark conductivity of p‐a‐SiCx:H and i‐a‐Si:H films were measured under varying temperatures (25–150 °C), and the activation energy for each condition was calculated using the Arrhenius equation.^[^ ^21^ ^]^ The composite layers and morphology of the a‐Si:H‐based VPDs were analyzed by field‐emission scanning electron microscopy (FE‐SEM; Sirion, FEI), and the specimens were prepared by focused ion beam milling. The dark and photo conductivities of the i‐a‐Si:H and the electrical characteristics of the p–i–n diode were measured using a probe station (MS‐TECH). To analyze the electrical properties of the p–i–n diode under illumination, the device was tested inside a dark‐shielded enclosure (MS‐Tech) equipped with an LED controller (BioLED light source control module, Mightex) and a 530‐nm light source (BLS‐LCS‐0470‐03‐22, Mightex). To analyze the resistance of each device, electrochemical impedance spectroscopy (EIS) was performed at a bias of 0.8 V using an electrochemical analysis system (CompactStat, Ivium Tech.). For repeated bending cycle tests, the photodiode flexibility was evaluated using a bending stage (M‐433, Newport) with various radii of curvature (5, 10, 15, and 20 mm).

## 3 Result and Discussion

**Figure 2:**
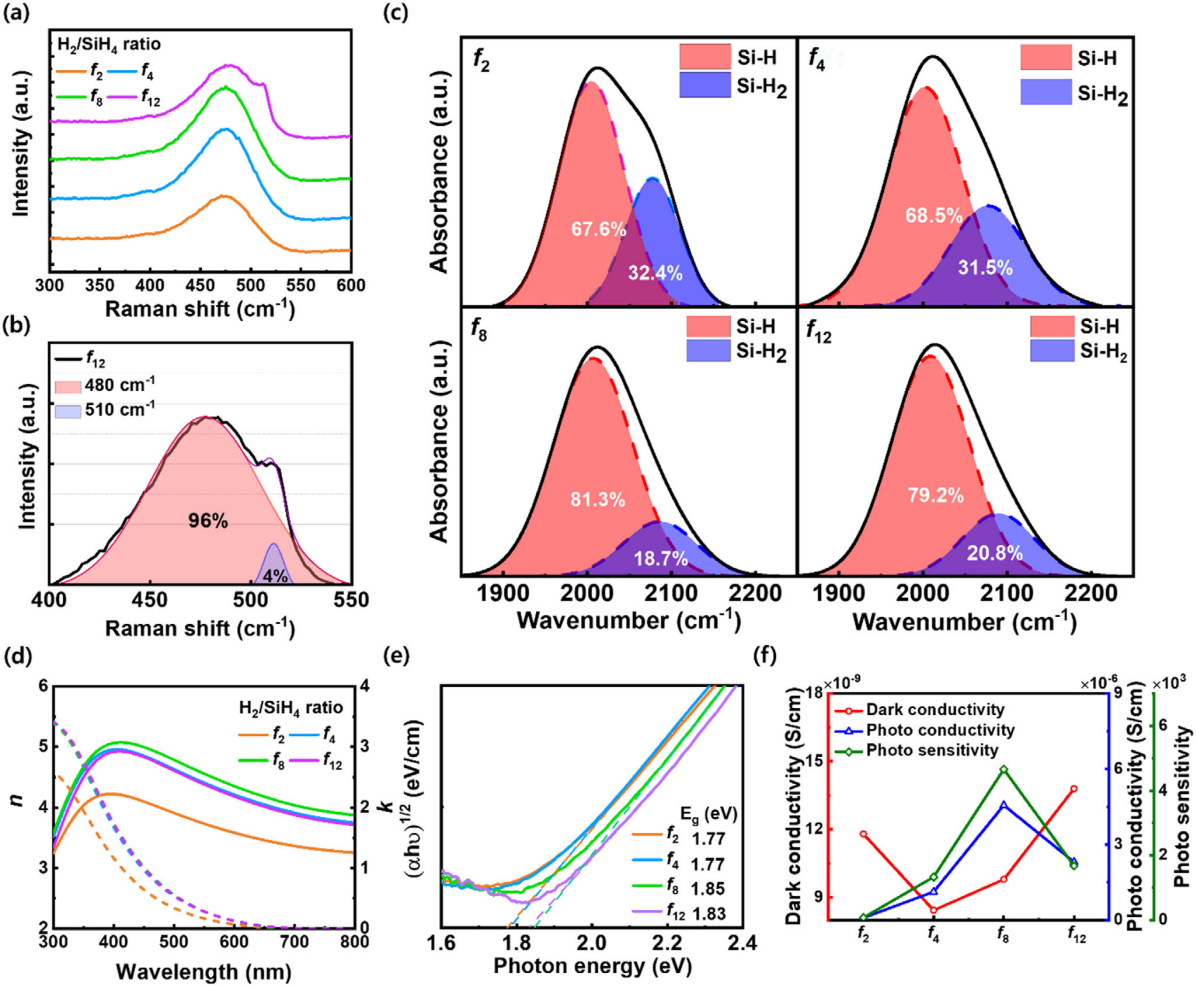
Material properties of intrinsic hydrogenated amorphous silicon (i‐a‐Si:H) prepared using PECVD at ultra‐low temperature of 90 °C: a) Raman spectra of i‐a‐Si:H films with varying the hydrogen/silane (*f*) flow ratio, b) deconvolution Raman spectra of *f*_12_ film, c) Fourier‐transform infrared (FT‐IR) spectra, d) refractive index (*n*) and extinction coefficient (*k*) spectra, e) Tauc plot for estimation of optical bandgap energies, and f) dark conductivity, photoconductivity, and photosensitivity of i‐a‐Si:H film with varying the *f* ratio.

While decreasing the deposition temperature of a‐Si:H can increase the hydrogen content, excessive hydrogen may lead to the formation of micro‐voids and unstable hydrogen bonds within the microstructure. These defects can cause structural instability and degrade the passivation quality, thereby making it challenging to achieve effective passivation during the low‐temperature PECVD process. Therefore, precise control of hydrogen passivation is essential to enhance film quality and ensure stable optical and electrical properties, which are crucial for the realization of high‐performance VPD devices. We systematically evaluated the physical properties of i‐a‐Si:H synthesized at an ultra‐low temperature of 90 °C. To investigate the microstructures of the i‐a‐Si:H films deposited at ultra‐low temperatures, the Raman spectra of each i‐a‐Si:H film were analyzed (**Figure** **2**a). For the hydrogen/silane gas flow ratio (*f*_x_ = H_2_/SiH_4_) ranging from *f*_2_ to *f*_8_, the amorphous matrix peak at 480 cm^−1^ remained dominant with no significant increase in additional peaks.^[^ ^22^, ^23^, ^24^ ^]^ However, at a dilution ratio of *f*_12_, a slight peak emergence was observed at ≈510 cm^−1^, suggesting the formation of nanocrystalline structures. To further clarify this structural evolution, the Raman spectra were deconvoluted (Figure 2b). Deconvolution analysis revealed that the i‐a‐Si:H film at *f*_12_ ratio consisted of an ≈96% amorphous matrix with an embedded 4% nanocrystalline fraction.

To precisely evaluate the hydrogen passivation and defect states of the i‐a‐Si:H films, FT‐IR spectra were analyzed for the i‐a‐Si:H films deposited with varying *f* ratios. As shown in Figure 2c, the peaks at 2000 and 2100 cm⁻¹ correspond to monohydride (Si─H) and dihydride (Si─H*_2_*) bonds, respectively.^[^ ^25^ ^]^ These represent the low and high stretching modes, respectively, and serve as powerful analytical tools for evaluating the fine structural details of hydrogenated amorphous silicon (a‐Si:H) networks based on the ratio of the microstructure stretching mode. The ratio of the monohydride bonds increases up to the *f*_8_ ratio in i‐a‐Si:H, whereas the high stretching mode decreases. The *f*_12_ ratio of i‐a‐Si:H shows a slight increase in dihydride bonds, which can occur with the formation of nanocrystalline Si domains within an amorphous matrix, leading to a higher density of dangling bonds with increased grain boundaries and unstable hydrogen bonding. The high stretching mode involves the expansion of the a‐Si network with strain, thereby leading to the formation of numerous defects within the film, that is, an increase in the number of voids and dangling bonds. Additionally, an excess concentration of unstable hydrogen promotes the formation of localized states within amorphous silicon, thereby enhancing trap‐assisted recombination, impeding carrier transport, and increasing the overall trap state density.^[^ ^26^, ^27^ ^]^ These defects enhance SRH recombination, which increases non‐radiative recombination in the absorber layer and deteriorates device performance. Additionally, to investigate effective hydrogen incorporation in i‐a‐Si:H, we derived the hydrogen concentration of each film from the stretch mode of the FT‐IR spectra (Figure S1, Supporting Information). The total hydrogen content in the i‐a‐Si:H films increase with higher hydrogen dilution ratios; notably, the proportion of monohydride bonding rises significantly up to the *f*_8_ condition, while dihydride bonding decreases. This transition in bonding configuration suggests that higher hydrogen dilution not only facilitates greater hydrogen incorporation but also promotes more effective passivation through the formation of stable Si─H monohydride bonds. These bonds are known to efficiently terminate dangling bonds without inducing excessive internal strain, unlike di‐ or poly‐hydride configurations, which tend to be structurally unstable and often correlate with micro‐void formation and electronic disorder. Therefore, monohydride‐rich hydrogenation can improve suppressing defect‐related phenomena which increased mid‐gap states and trap‐assisted recombination ultimately minimizing SRH recombination losses and enhancing the electronic quality of the films.

To investigate the optical properties of the i‐a‐Si:H films as a function of hydrogen passivation quality, we analyzed the refractive index (n) and extinction coefficient (k) using spectroscopic ellipsometry with varying *f* ratios. As shown in Figure 2d, the n values exhibit a gradual increase across the entire wavelength range as the *f* ratio increases to 8, followed by a decrease at the *f*_12_ ratio. The n value of semiconductor thin films tends to increase as the material density increases and the void fraction decreases. This trend is consistent with the FT‐IR results, which indicate that the most effective hydrogen passivation and minimal film strain occur at the *f*_8_ ratio of the i‐a‐Si:H film, which correlates with the highest n values. Thus, the i‐a‐Si:H film deposited at *f*_8_ can be inferred to have a higher amorphous matrix density owing to the reduction in defect states. The k values of *f*_4_, *f*_8_, and *f*_12_ are similar; however, *f*_2_ exhibits lower values, which leads to a lower absorption by the film compared with the other films. To investigate the light absorption capacity of each thin film and the band gap (E_g_) characteristics of the microstructures, the E_g_ values of the i‐a‐Si:H films were determined using Tauc's method. As shown in Figure 2e, E_g_ shows a slight increase with an increase in the *f* ratio. The electronic band structure of a‐Si:H is affected by the band tail and defect states inherent to its amorphous nature. A high density of defects in the localized states can extend the energy states toward the band edges, resulting in a reduction in E_g_. Conversely, sufficient hydrogen passivation reduces the density of localized states and band‐tail states, thereby sharpening the optical absorption edge and effectively increasing E_g_. The FT‐IR results indicate that the most effective hydrogenation occurs at *f*_8_, correlating with the highest observed E_g_ under these conditions.

To evaluate the electrical and optoelectronic performances of the i‐a‐Si:H films, the dark conductivity and photoconductivity were measured by varying the *f* ratio. The dark conductivity decreased up to *f*_4_ before increasing again, whereas the photoconductivity increased up to the *f*_8_ ratio and then declined to the *f*_12_ ratio. In applications such as photodiodes, where i‐a‐Si:H serves as the photoactive absorber layer, performance can be assessed using photosensitivity, which is defined as the ratio of photoconductivity to dark conductivity. The higher photosensitivity observed for the *f*_8_ device is attributed to its longer carrier lifetime confirmed on transient photovoltage measurements (Figure S2, Supporting Information), arising from superior defect passivation, rather than from an increased electron–hole generation rate.^[^ ^28^ ^]^ The highest photosensitivity was observed at *f*_8_ of the i‐a‐Si:H film, which agreed with the results of the FT‐IR analysis, indicating that a lower Si‐H_2_ bond content minimizes defect state formation. A decrease in the number of defects effectively suppresses non‐radiative recombination pathways, thereby enhancing the optoelectronic performance of the film. Consequently, more photogenerated carriers reach the electrodes without recombination, leading to superior device performance.

**Figure 3:**
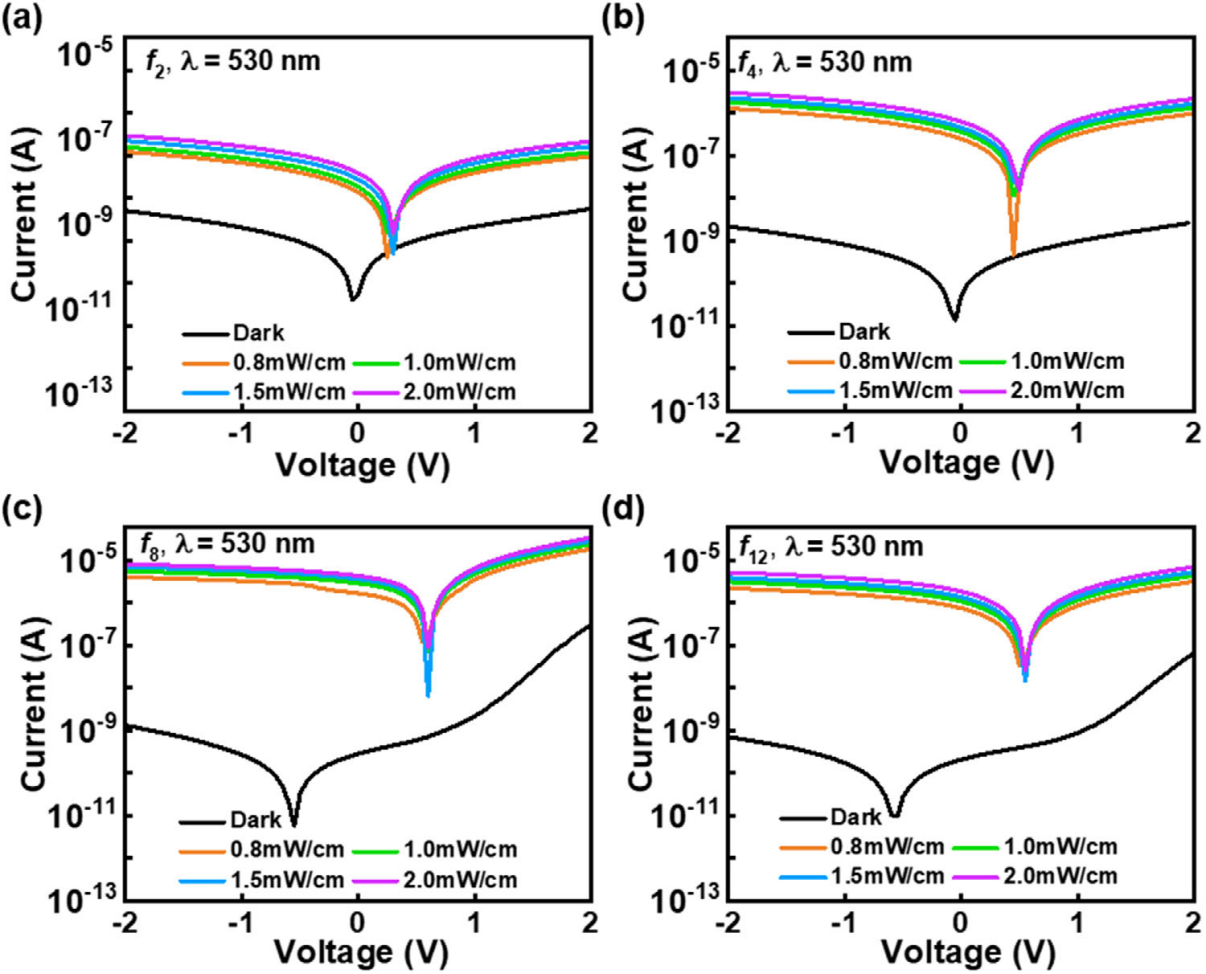
Current–voltage (*I─V*) characteristics of the photodiode measured under varying light intensities at a wavelength of 530 nm: a) *f*_2_, b) *f*_4_, c) *f*_8_, and d) *f*_12_ ratio.

In a‐Si:H‐based photoelectronic devices, the quality of the i‐layer, which primarily absorbs light and facilitates the separation of photoexcited carriers via the built‐in potential, plays a critical role in determining device performance. The quality of the i‐layer is significantly influenced by its microstructure, which contains numerous dangling bonds and defect states. Based on the thin‐film properties of i‐a‐Si:H investigated in Figure 2, a‐Si: H‐based flexible VPDs were prepared to investigate their photodiode characteristics. Figure 1j presents the schematic structure and cross‐sectional FE‐SEM image of the a‐Si:H‐based VPDs. The FE‐SEM image shows that the a‐Si:H layers and transparent conductive oxide layers are distinctively deposited on their sites, and the p–i–n VPD is successfully fabricated on the flexible PEN substrate via lift‐off lithography. To investigate the impact of hydrogen passivation in i‐a‐Si:H deposited at ultra‐low temperature of 90 °C, we measured the current (I)–voltage (V) characteristics of a‐Si:H‐based VPDs by varying the hydrogen/silane gas flow ratio from *f_2_* to *f*_12_. As shown in **Figure** **3**, as the *f* ratio increases, a notable decrease is observed in the dark current (I_d_), indicating that the increased hydrogen dilution ratio during deposition enhances the passivation of defects, thereby minimizing the pathways for charge recombination or leakage current. The photocurrent (I_P_) at each *f* ratio was measured under varying light intensities (0.8–2.0 mW cm^−2^) at a wavelength of 530 nm, which serves as a representative measurement wavelength within the visible spectrum for a‐Si:H.^[^ ^29^ ^]^ The I_P_ of the a‐Si:H‐based VPDs exhibits a linear increase across all *f* ratios with increasing light intensity, and the highest photocurrent value is observed at *f*_8_. To quantitatively compare the performance of the VPDs across different f ratios, the photocurrent‐to‐dark current ratio (PDCR), which is an index of the optical detection performance, was calculated using Equation (1):

(1) $$\mathrm{PDCR}\;=\frac{I_{p}-I_{d}}{I_{d}}$$

The detailed PDCR values for each *f* ratio are listed in **Table** **3**. At −1 V and 1 mW cm^−^^2^, the PDCR value for the *f*_8_ ratio reaches the highest value of 3391, and the higher PDCR indicates that the photocurrent is substantially larger than the dark current relative to the magnitude of dark current, which suggests an improvement in the sensitivity of the photodetector.^[^ ^30^ ^]^

**Table 3:** photocurrent‐to‐dark current ratio values of each flexible visible light photodetectors (VPDs).

	*f*_2_	*f*_4_	*f*_8_	*f*_12_
PDCR	16	509	3391	3267

**Figure 4:**
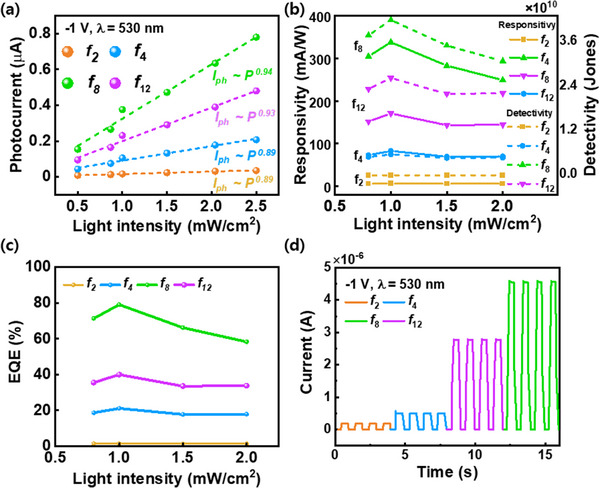
a) Photocurrent variation, b) responsivity and Specific detectivity, c) external quantum efficiency (EQE) results of a‐Si:H based VPDs with varying the incident light intensity at −1 V and a wavelength of 530 nm, and d) response time measurements showing on/off current characteristics.

For further investigating the performance of VPDs with varying *f* ratios, the I_P_ values of each *f* ratio according to the light intensity at −1 V were analyzed (**Figure** **4**a). The relationship between photocurrent and light intensity follows a power law, as shown in Equation (2).

(2) $$I_{P}=AP^{\alpha }$$

where A is the scaling constant, *P* is the incident light intensity, and α is the power law exponent. As the light intensity increases, the extrapolated slope provides insight into the rate at which the photocurrent increases. When charge carriers are efficiently transported and collected without significant losses caused by defect‐related recombination and trapping, the α value approaches 1. The α values are enhanced with increment from *f*_2_ to *f*_8_ (0.89 to 0.94), signifying optimal carrier transport with minimal hindrance.^[^ ^31^, ^32^ ^]^ Responsivity (*R*) is a critical parameter that defines the efficiency of a photodiode in converting incident optical signals into electrical output, and is calculated using Equation (3):

(3) $$R=\frac{I_{P}-I_{d}}{P_{\lambda }\times S}$$

where *P*_λ_ is the incident optical power density and *S* is the active area of the VPDs. Specific Detectivity (*D* ^*^) is a widely used metric for evaluating the sensitivity of photodetectors and can be evaluated using Equation (4):^[^ ^33^ ^]^

(4) $$D^{*}=\frac{R\sqrt{S}}{\sqrt{2eI_{d}}}\;$$

where e denotes the elementary charge. Figure 4b shows the *R* and *D* ^*^ values of each VPD under varying light intensities. The R value of the *f*_8_ device is ≈338 mA W^−1^ under a bias of −1 V and incident light intensity of 1 mW cm^−2^. Despite the extremely low deposition temperature of 90 °C, this performance is comparable to the 350 mA W^−1^ value reported in a previous study on devices deposited at a higher temperature of 250 °C, implying that the precise control of hydrogen passivation in amorphous networks is effective. In addition, the *D* ^*^ value of the *f*_8_ ratio is 4.23 × 10^10^ Jones. Compared with the typical *D* ^*^ value of 10^10^–10^11^ Jones reported for amorphous Si, this result demonstrates the competitiveness of our flexible VPDs.^[^ ^34^ ^]^

The external quantum efficiency (EQE) represents the ratio of the number of charge carriers generated to the number of incident photons, and serves as a key parameter of the photoelectric conversion efficiency of the device.^[^ ^35^ ^]^ The EQE was calculated using Equation (5)

(5) $$\;\mathrm{EQE}\;=R\frac{h\lambda }{e}$$

where *h* and λ are Planck's constant and wavelength, respectively. The calculated EQE values for various light intensities are presented in Figure 4c. Under the *f*_8_ ratio at a bias of −1 V, the device achieves an EQE of 83%. The results confirm that the p‐i‐n diode maintains robust performance metrics, including R, *D* ^*^, and EQE, even under low‐temperature (90 °C) and low‐voltage (−1 V) conditions, without significant degradation. Furthermore, the *R*, *D* ^*^, and EQE peaks at 1 mW cm^−2^ gradually decrease thereafter. This decline is attributed to the gradual saturation of the photodetector within the structure.^[^ ^36^ ^]^ The superlinear behavior observed under low light intensities is attributed to the gradual filling of trap states, which enables electrons to move more freely, thereby enhancing the current flow. As the light intensity increases, the impact of SRH recombination diminishes.^[^ ^37^ ^]^ Moreover, owing to the finite density of trap states, the number of captured carriers eventually saturate or decrease, leading to a pronounced reduction in the photoresponsivity and a concurrent increase in trap‐assisted recombination.^[^ ^38^ ^]^ The response time, an important parameter for photodiodes, defines the duration required for the device to respond to variations in the incident light intensity. It is typically characterized by the rise time (τ_r_) and fall time (τ_f_), representing the time taken for the output signal to increase from 10% to 90% and decrease from 90% to 10% of its maximum value, respectively.

A short response time is highly desirable for applications requiring high‐speed signal processing. As shown in Figure 4d, the on/off current ratio under the *f*_8_ ratio is the highest among *f* ratios. Although the response time is relatively low compared to that of other photodiodes, the distinct and well‐defined on/off current ratio ensures superior signal discrimination and precise optical signal detection in practical applications. The pronounced difference in current between the on and off states facilitates accurate signal differentiation, thereby enabling efficient and stable data processing for high‐performance optoelectronic systems.^[^ ^39^, ^40^ ^]^ To further assess the practical performance of our photodetectors, we quantitatively analyzed their linear dynamic range (LDR), which represents the range of incident light intensities over which the device maintains a linear photo‐response. As illustrated in Figure S3 (Supporting Information), the device fabricated under the *f*_8_ condition exhibited the broadest linear regime, achieving an LDR of 76.4 dB, which is significantly higher than those of other conditions. This result confirms the superior photo‐detection linearity and signal fidelity of the *f*_8_‐based device, even at low irradiance levels. The broad LDR reinforces the applicability of our ultra‐low‐temperature processed a‐Si:H photodetectors in real‐world environments requiring stable and wide‐range optical sensing. Scalable and uniform thin‐film deposition over large areas is a key requirement for industrial viability and cost‐effective device integration. Large‐area deposition tests on 20 × 20 cm^2^ substrates (Figure S4, Supporting Information) demonstrated excellent thickness uniformity of the *f*_8_ a‐Si:H films, supporting the scalability of the ultra‐low‐temperature PECVD process.

**Figure 5:**
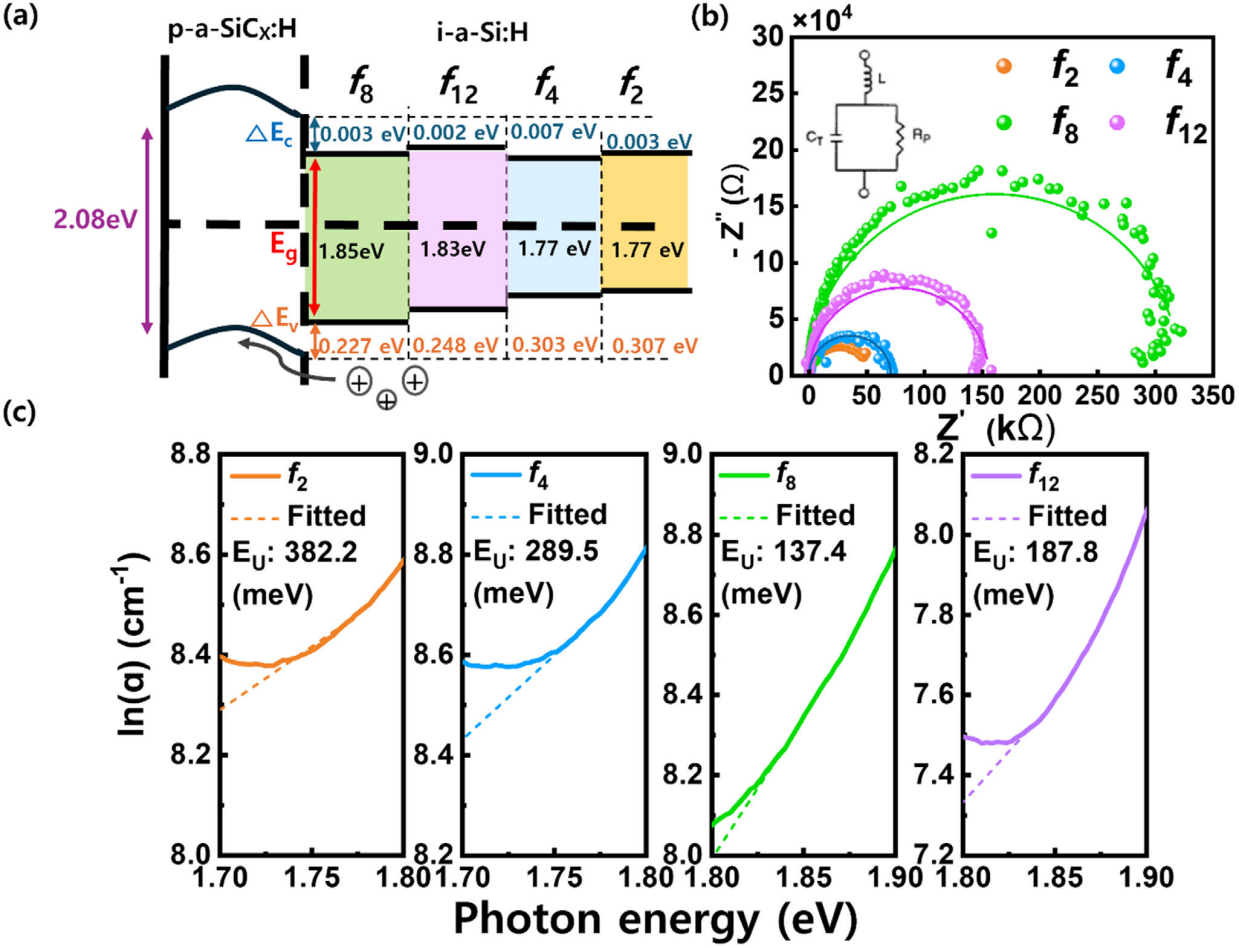
a) Bandgap Energy band diagram of the p‐i interface, illustrating valence band offset and charge transport characteristics, b) electrochemical impedance spectroscopy (EIS) results showing shunt resistance to evaluate leakage current and alternative conduction paths, and c) logarithmic absorption coefficient of i‐a‐Si:H films with varying *f* ratios for calculation of Urbach energy (E_U_).

The superior device performance of *f*_8_ is shown in Figures 3 and 4. To provide a clear analysis from a device‐physics perspective, we investigated the energy‐band diagram to elucidate the underlying mechanisms (**Figure** **5**a). The energy band diagram was derived using AFORS‐HET simulation (Figure S5, Supporting Information), and the experimental parameters such as E_g_ and the activation energies of p‐a‐SiCx:H and i‐a‐Si:H are listed in **Table** **4**, and detailed simulation parameters are listed in Table S1 (Supporting Information). In the energy band diagram, the valence band offset (VBO) values of p/i interface are 0.227, 0.248, 0.303, and 0.307 eV in *f*_8_, *f*_12_, *f*_4_, and *f*_2_, respectively. Also, the conduction band offset (CBO) values of p/i interface are 0.003, 0.002, 0.007, and 0.003 eV in *f*_8_, *f*_12_, *f*_4_, and *f*_2_, respectively. i‐a‐Si:H films exhibit similar CBO values so that the main p/i interfacial recombination process dominantly occurs by VBO. The VBO of *f*_8_ ratio is lower than that of other ratios of the i‐a‐Si:H films, and a low VBO suggests that photogenerated carriers, particularly holes, experience reduced recombination at the p/i interface, thereby facilitating efficient hole collection in the p‐layer.^[^ ^41^ ^]^ Notably, the carrier recombination kinetics investigated using the energy‐band diagram suggest that the *f*_8_ ratio of i‐a‐Si:H has the highest potential to function as an optimal VPD. To further investigate the recombination kinetics in VPDs with varying hydrogen content of the i‐a‐Si:H film, EIS was performed under dark conditions (Figure 5b). The Nyquist plot is closely related to the series contact resistance and shunt‐related resistance of the material and interfaces, which are considered as the resistance of the alternate or parallel paths for leakage current conduction.^[^ ^42^ ^]^ The plotted semicircle shows that the *f*_8_ ratio is the largest among the H_2_/SiH_4_ ratios with a high real impedance (Z’), indicating the possibility of the lowest leakage point. Similar to the analysis of the energy band diagram, the resistance analysis also demonstrates the improved properties of *f*_8_ ratio, facilitating efficient carrier separation and transport owing to the high film quality with proper hydrogenation and a lower VBO value.^[^ ^43^, ^44^ ^]^ To assess the impact of i‐a‐Si:H on the performance of the VPDs, particularly in terms of the photoelectric effect, we measured the Urbach energy (E_U_) of i‐a‐Si:H as a function of the *f* ratio (Figure 5c). E_U_ was determined from the reciprocal slope of the linear extrapolation of the logarithmic absorption coefficient.^[^ ^45^ ^]^ Among the intrinsic layers, the *f*_8_ ratio exhibited the lowest E_U_, indicating a reduced density of localized states, as corroborated by the FT‐IR analysis results. The E_U_ value quantifies the extent of bandtail absorption, which arises from the matrix disorder and is closely linked to parasitic absorption and charge trapping. A higher E_U_ corresponds to an expanded band tail, where the absorbed energy and trapped charges dissipate through non‐radiative recombination, ultimately degrading the photoelectric conversion efficiency. Therefore, achieving an optimal hydrogen passivation in a‐Si:H is critical for enhancing the performance of VPDs.

**Table 4:** Bandgap energy (E_g_) and activation energy (E_a_) of p‐a‐SiCx:H and i‐a‐Si:H films with varying *f* ratios.

	E_g_ [eV]	E_a_ [eV]
p‐a‐SiC_x_:H	2.08	0.88
*f*_2_	1.77	0.61
*f*_4_	1.77	0.72
*f*_8_	1.85	0.89
*f*_12_	1.83	0.74

To further evaluate the electronic trap states in the a‐Si:H films, we conducted space‐charge‐limited current (SCLC) measurements and extracted the density‐of‐states (DOS) distribution within the energy range below the conduction band edge. As shown in Figure S6 (Supporting Information), the *f*_2_ and *f*_4_ samples exhibited significantly higher trap densities, particularly in the shallow energy region (E_C_ – E < 0.1 eV), indicating a large concentration of localized states that hinder efficient carrier transport. These shallow traps are often associated with unsaturated dangling bonds and structural disorder, both of which are exacerbated under low hydrogen dilution conditions. In contrast, the *f*_8_ and *f*_12_ films revealed substantially lower and more uniform DOS profiles across the analyzed energy range, reflecting a reduced density of electrically active trap states. Notably, the *f*_8_ sample exhibited the lowest overall trap density, which correlates well with its lowest Urbach energy and highest device performance metrics. An optimal hydrogenation effectively mitigates the formation of shallow defects and promotes superior electronic passivation, thereby enhancing the transport properties and long‐term operational stability of the a‐Si:H films. To provide rigorous theoretical support for our experimental findings, we conducted numerical simulations to evaluate the distribution of defect states within the bandgap of i‐a‐Si:H films using the AFORS‐HET simulation software (Figure S7, Supporting Information). Experimentally determined optical bandgaps, activation energies, and E_U_ served as critical input parameters for modeling films deposited under different hydrogen dilution ratios. The simulations explicitly accounted for both exponential tail states near the conduction and valence band edges, as well as localized mid‐gap defect states, enabling quantitative assessment of the total effective DOS. Among the simulated conditions, the film deposited at the optimal hydrogen dilution ratio (*f*_8_) demonstrated the narrowest tail‐state distribution and the lowest effective DOS, consistent with its experimentally measured minimal Urbach energy (137.4 meV) and superior optoelectronic device performance. Conversely, films prepared with lower hydrogen dilution ratios (*f*_2_ and *f*_4_) exhibited broader tail‐state distributions and significantly increased mid‐gap DOS, reflecting a higher degree of structural disorder that facilitates trap‐assisted recombination processes and deteriorates charge transport. This correlation is further substantiated by Figure S8 (Supporting Information), where the calculated effective DOS profiles clearly illustrate the lowest mid‐gap state density for the *f*_8_ condition, affirming its enhanced electronic quality. Overall, these simulation results unambiguously establish a robust correlation between optimized hydrogenation conditions, reduced electronic disorder, and improved device performance, thereby confirming that effective hydrogen incorporation at ultra‐low temperatures substantially minimizes defect‐related states and promotes efficient carrier transport in i‐a‐Si:H films.

**Figure 6:**
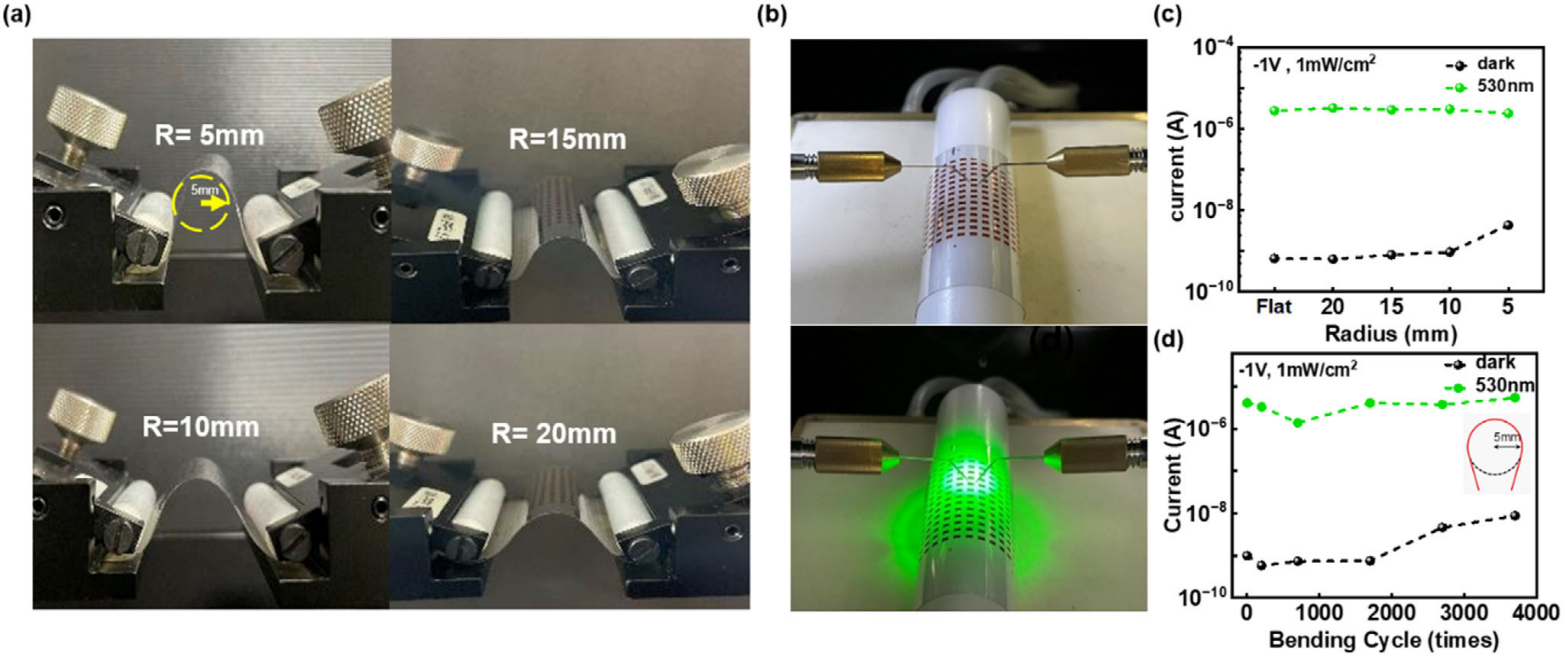
a) The images of bending test system with various curvature radii (20, 15, 10, and 5 mm), and dark and photocurrent measurements of flexible VPDs for mechanical reliability: b) the images of bended measurement system, c) 2700 bending cycle results with varying the bending radius, and d) repeated bending cycle test results under a 5 mm bending radius.

The mechanical stability of flexible VPDs under bending conditions is essential for their practical integration into flexible multifunctional optoelectronic systems. The fabricated VPDs exhibited excellent flexibility and robustness against bending tensile stresses. We investigated the mechanical reliability of the flexible VPDs using repeated bending cycling tests at various radii (5, 10, 15, and 20 mm) with *f*_8_ ratio; mechanical bending tests were performed with or without incident light (**Figure** **6**a,b). The flexible VPD exhibits similar and stable dark current states from 20 to 10 mm of bending radius, and the bending radius of 5 mm shows a slight increase in the dark current with a stable photoresponse and without cracks or tearing at all curvature radii ranging up to 2700 cycles, as shown in Figure 6c. This indicates that except under extreme conditions, mechanical bending had no significant impact on the photosensing performance, and the mechanical reliability of VPD can be considered excellent. In addition, to investigate the mechanical performance under extreme loading conditions, repeated bending cycling tests of up to 3700 cycles at a bending radius of 5 mm were conducted (Figure 6d). The device maintained stable on/off operation even after 2000 continuous bending cycles, with performance degradation observed only beyond this point. These performances have outstanding mechanical reliability while maintaining the photosensing capability even when using inorganic materials constituting the VPDs, which is higher than that reported in previous studies.^[^ ^46^ ^]^ Additionally, we investigated various stability tests for practical applications of the flexible VPDs. Long‐term environmental exposure is a critical factor affecting the operational reliability of flexible optoelectronic devices. The flexible VPD retained its optoelectronic performance after 6 months of ambient storage (25 °C, 55% RH), with a reduced dark current and enhanced PDCR, demonstrating excellent long‐term environmental stability (Figure S9, Supporting Information). Also, stable dark and photocurrent characteristics were maintained up to 2000 bending cycles, confirming the device's robust mechanical durability with long‐term stability. Photodetectors integrated into flexible systems often experience thermal stress during operation or packaging, necessitating reliable thermal performance. Thermal exposure up to 80 °C for 8 h resulted in negligible degradation in both dark and photocurrent characteristics, confirming the device's thermal stability under elevated temperatures (Figure S10, Supporting Information). a‐Si:H is known to suffer from light‐induced metastable defects (Staebler–Wronski effect), which can degrade device performance under prolonged illumination. Under continuous illumination for 3326 s at both 1 and 30 mW cm^−2^, the device showed minimal photocurrent reduction, indicating strong resistance to light soaking (Figure S11, Supporting Information). To verify the competitiveness of flexible VPDs based on a‐Si:H synthesized at ultra‐low temperature through effective hydrogenation, we compared the responsivity of our device with that of representative rigid and flexible photodetectors reported across a range of material systems, including hybrid perovskites, metal oxides, and Si‐based semiconductors (Figure S12, Supporting Information). Our study exhibits a relatively high responsivity, positioning itself within the upper range of reported flexible photodetectors, despite being synthesized without any post‐deposition annealing. These results confirm that our approach achieves competitive performance under significantly milder processing conditions, underscoring its viability for future applications in flexible optoelectronics. These results demonstrated that the photodetector synthesized under the *f*_8_ ratio, with hydrogen passivation optimized through a low‐temperature process at 90 °C, exhibited comparable optoelectronic performance to those of p‐i‐n diodes fabricated via conventional high‐temperature processes. Simultaneously, the device exhibited superior mechanical flexibility and stability. This underscores the potential of the low‐temperature direct lift‐off method and optimized hydrogen passivation for the development of ultraflexible and wearable optoelectronic devices.

## 4 Conclusion

We developed ultra‐low‐temperature (90 °C) PECVD‐deposited a‐Si:H films for application in VPDs. By systematically varying the hydrogen‐to‐silane gas flow ratio, we identified an optimal dilution ratio (*f*_8_) that minimized the defect states and improved passivation, as confirmed via Raman and FT‐IR analyses. The optimized film exhibited a higher refractive index and improved bandgap characteristics, leading to enhanced optical absorption and charge‐transport efficiency. Photodiodes with the optimized a‐Si:H layer demonstrated high photosensitivity, low dark current, and efficient charge carrier collection. Under visible illumination (λ = 530 nm), they achieved a responsivity of 338 mA W⁻¹, specific detectivity of 4.23 × 10^10^ Jones, and EQE of ≈83%. Additionally, flexible photodetectors fabricated using this material exhibited excellent mechanical stability under repeated bending cycles, maintaining their performance even after ≈2000 cycles. Furthermore, they demonstrated resilience under extreme bending conditions, including wrapping around a small rod (*r* = 5 mm), demonstrating their suitability for flexible optoelectronic applications. These findings provide a significant step toward the development of high‐performance, low‐temperature‐processed a‐Si:H photodetectors with potential applications in flexible and transparent sensing devices.

## Conflict of Interest

The authors declare no conflict of interest.

## Author Contributions

Y.‐J.J. contributed to the experiments, data analysis, and manuscript preparation as the first author; K.‐j.H., H.‐W.J., Y.‐H.K., and J.‐W.Y. contributed to the experiments and data analysis as co‐second authors; W.‐I.P. contributed to experiment management and conceptualization as the corresponding author; S.‐W.C. contributed to conceptualization and manuscript review; J.‐D.K. contributed to experiment management, conceptualization, review, and funding acquisition.

## Supporting information



Supporting Information

## Data Availability

The data that support the findings of this study are available from the corresponding author upon reasonable request.
