# Supplementary material for: Tailoring Hydrogenation to Enhance Defect Suppression and Charge Transport in Hydrogenated Amorphous Silicon for Flexible Photodetectors
Source: Adv Sci (Weinh). 2025 Jun 23;12(31):e04199. doi: 10.1002/advs.202504199 (PMC12376608; doi:10.1002/advs.202504199)
Supplement: Supplementary file 1 — Supporting Information [file ADVS-12-e04199-s001.docx]

**Supporting Information**

**Tailoring hydrogenation to enhance defect suppression and charge transport in hydrogenated amorphous silicon for flexible photodetectors**

*Ye-ji Jeong^1,2^, Kyeong-jin Hyun ^1,2^, Hee-Won Jang^1,3^, Jong-won Yun^1^, Yong-Hun Kim^1^, Woon-Ik Park^2,^***, Soo-Won Choi^1,^** *and Jung-Dae Kwon^1,^**

^1^ Energy & Environment Materials Division, Korea Institute of Materials Science, Changwon, Gyeongnam 51508, Republic of Korea.

^2^ Department of Materials Science and Engineering, Pukyong National University, Busan 48513, Republic of Korea

^3^ Department of Materials Science and Engineering, Pusan National University, Busan 46241, Republic of Korea

E-mail: thane0428@pknu.ac.kr (W.-I Park)

tndnjs6793@unist.ac.kr (S.-W choi)

jdkwon@kims.re.kr (J.-D kwon)

**Figure S1.** Hydrogen concentration (N_H_) and atomic percent Hydrogen (C_H_) of i-a-Si:H calculated from the absorption coefficient of silicon-hydrogen stretch mode.


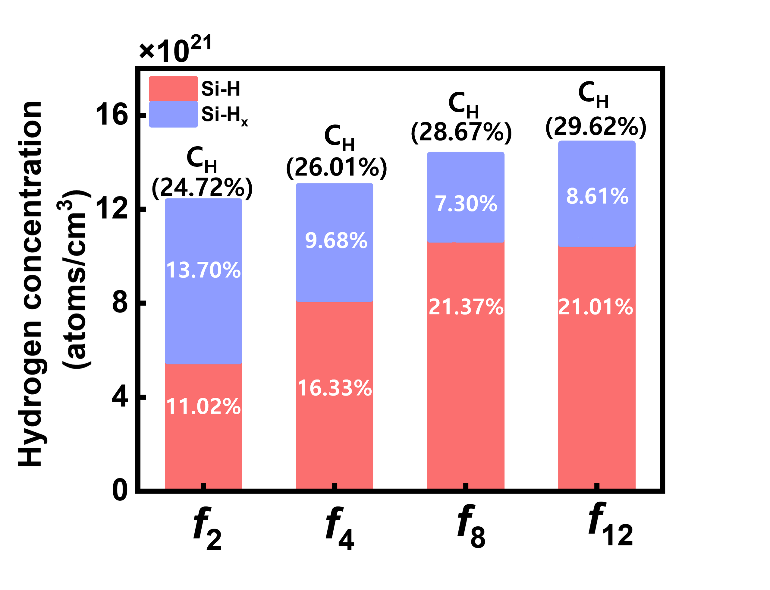


**Figure S2.** Transient photovoltage measurements of i-a-Si:H varying the *f* ratio.


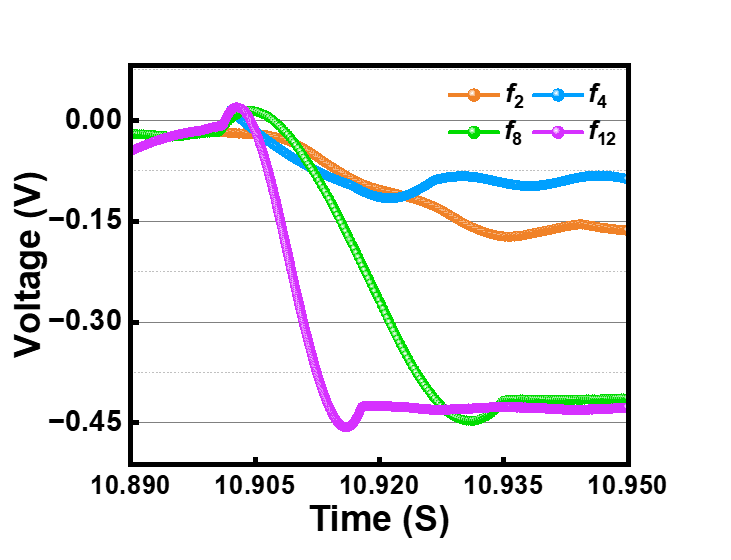


**Figure S3.** Current density measurement of a-Si:H-based flexible VPDs with varying the incident light intensity for calculation of linear dynamic range (LDR).


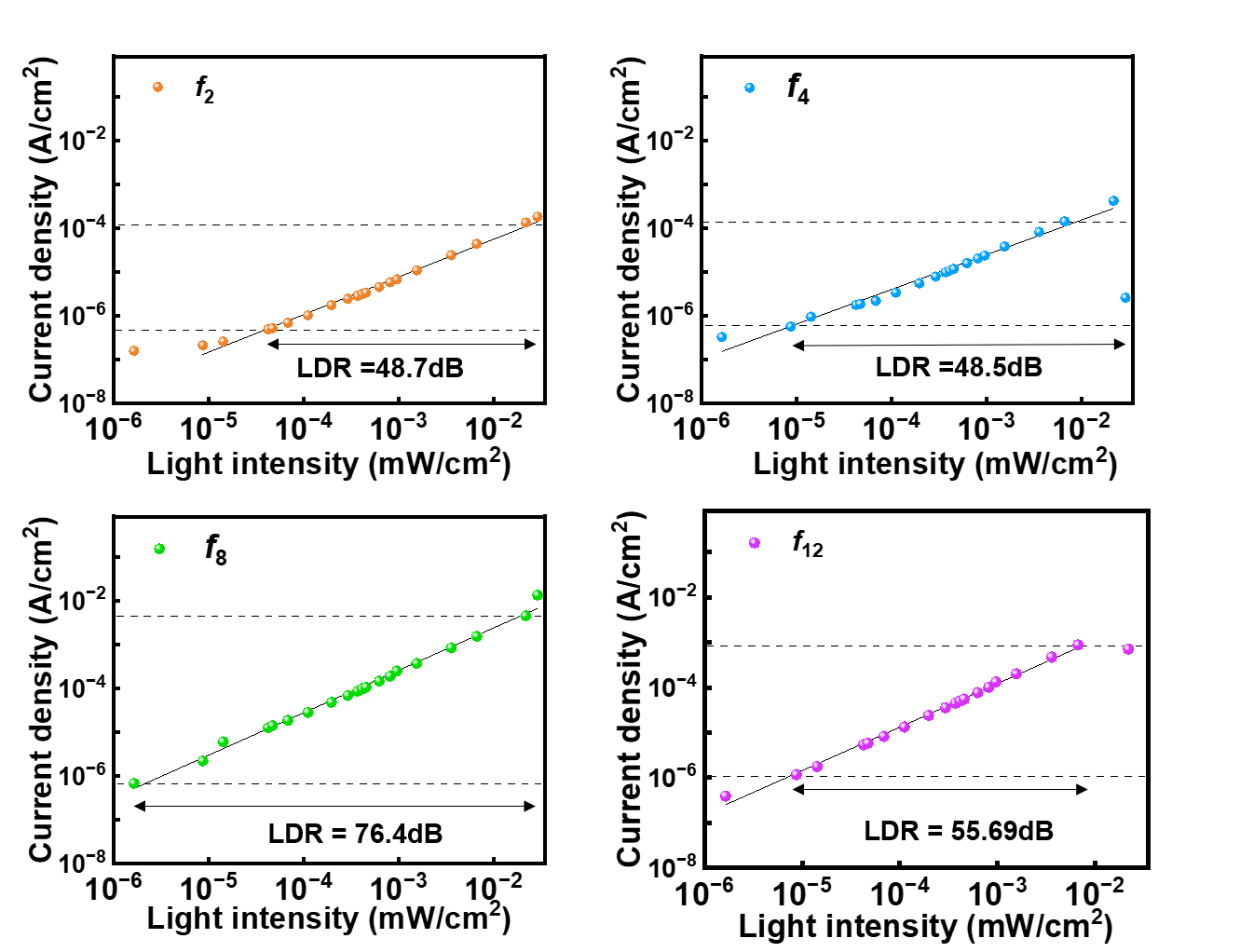


**Figure S4.** Thickness deviation of i-a-Si:H films (*f*_8_ ratio) deposited on 20 x 20 cm^2^ soda-lime glass, evaluated at 45 uniformly distributed points for target thicknesses of 100 nm and 400 nm.


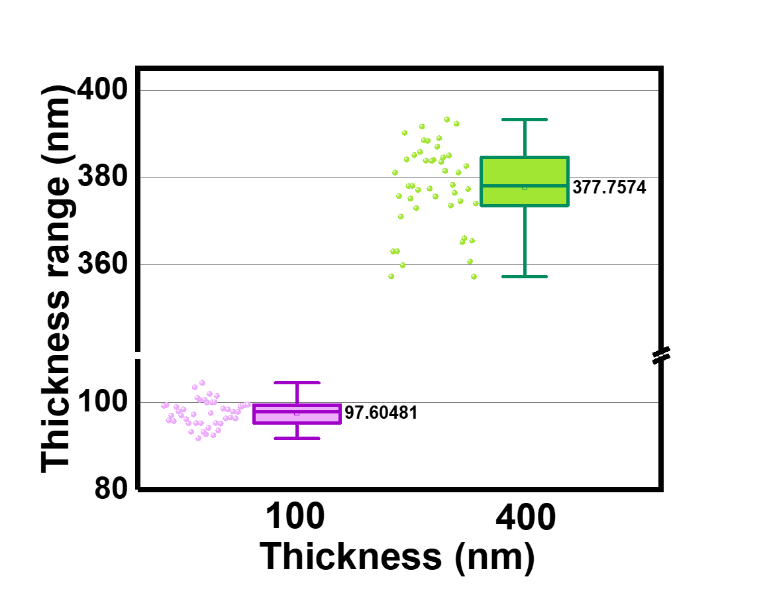


**Figure S5.** Energy band diagram of p/i interface using AFORS-HET simulation with varying the *f* ratio in i-a-Si:H film.


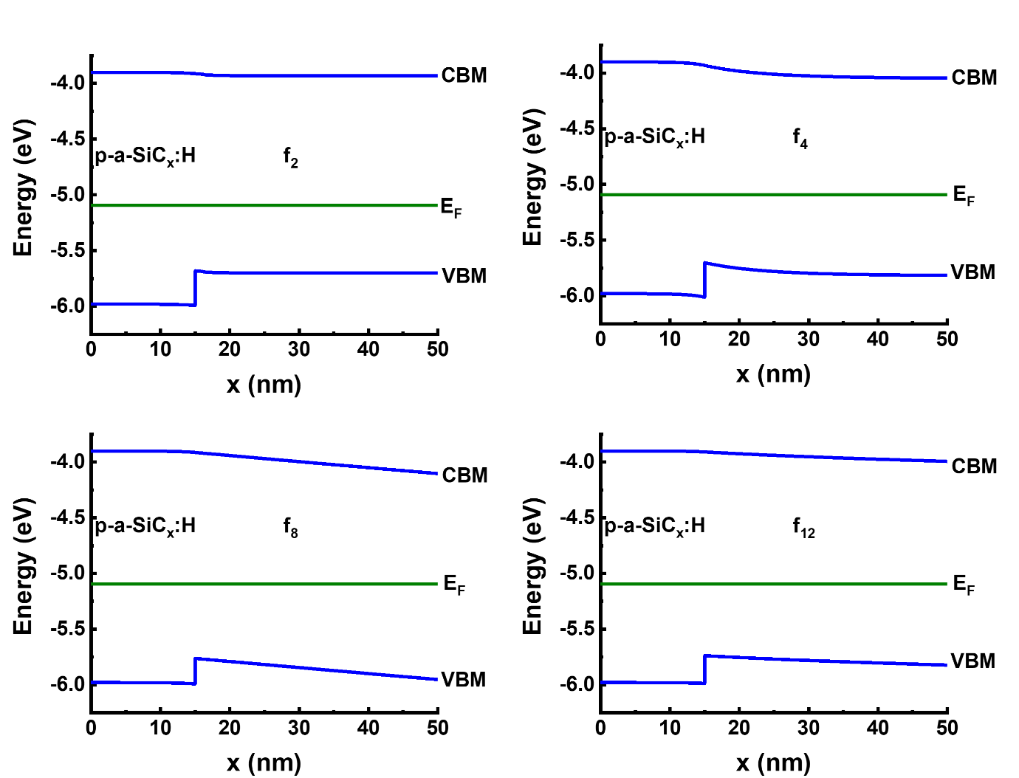


**Figure S6.** Energy-resolved density of states (DOS) profiles of i-a-Si:H films extracted from space-charge-limited-current (SCLC) measurements under various hydrogen dilution conditions.


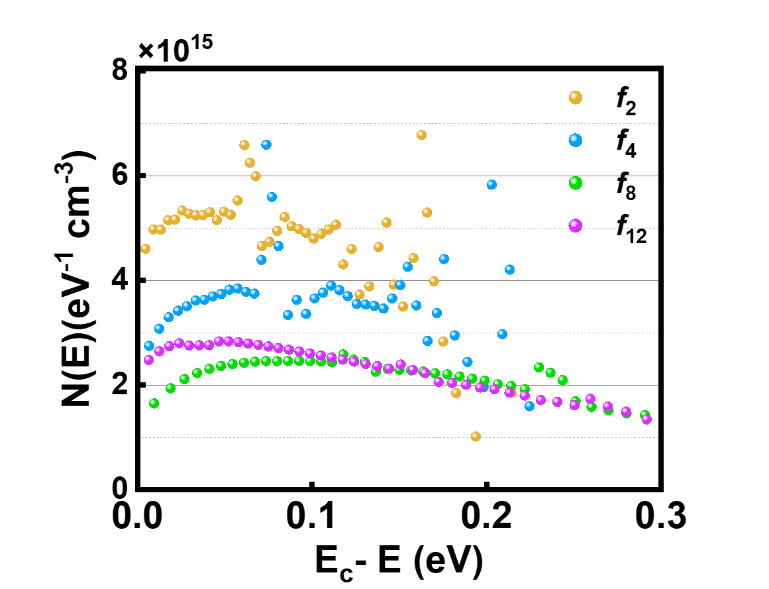


**Figure S7.** Energy distribution of defect states in intrinsic a-Si:H films with varying hydrogen dilution ratios calculated using AFORS-HET simulation.


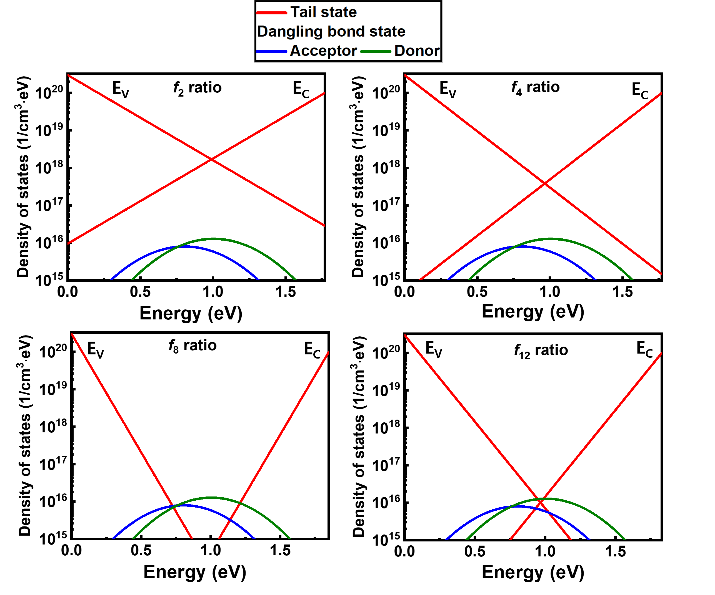


**Figure S8.** Effective density of states in defect states for i-a-Si:H films corresponding Figure S7.


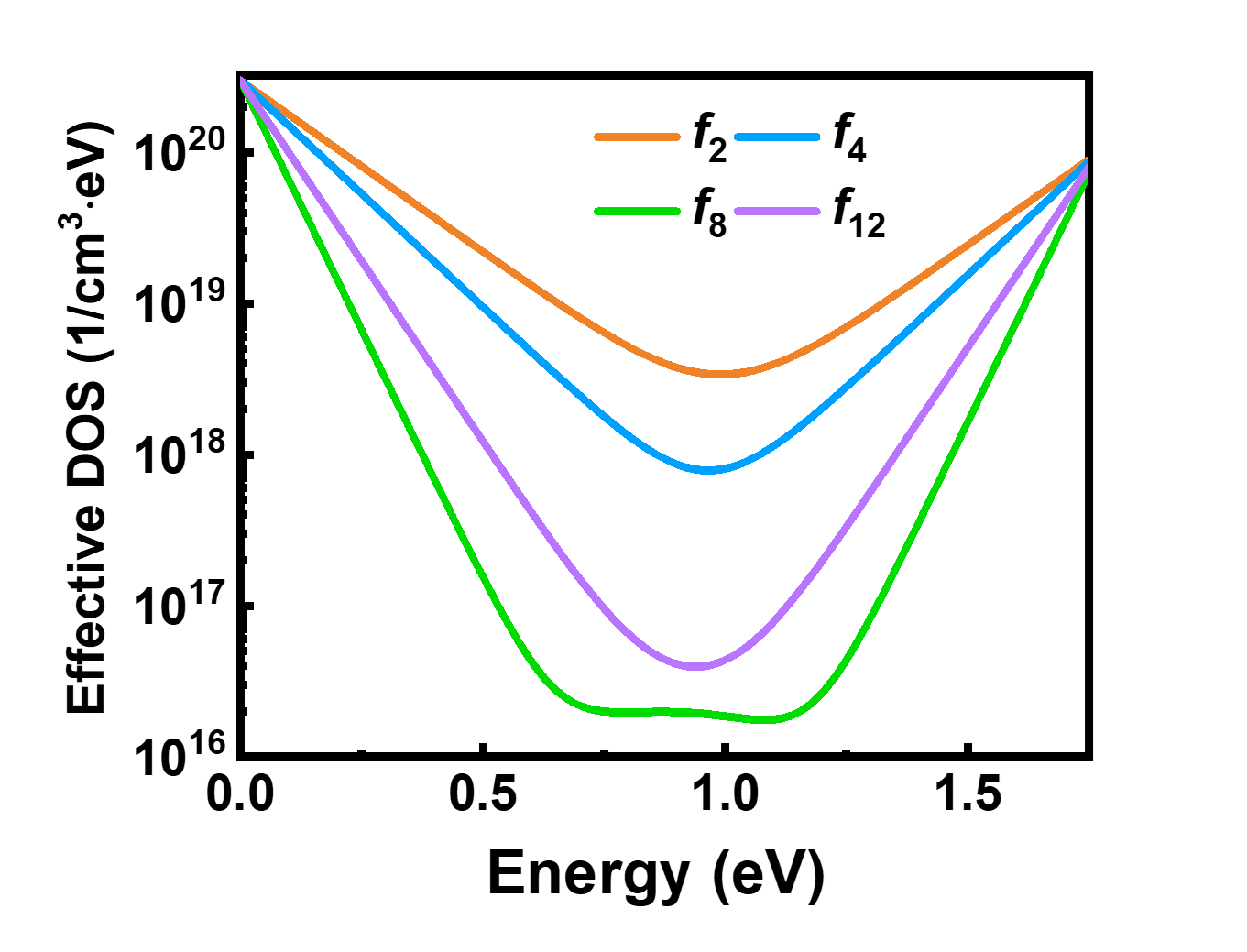


**Figure S9.** Dark and photocurrent characteristics of flexible VPD (f_8_ ratio) under repeated bending cycles, comparing pristine samples (circular markers) and those stored for six months at 25 °C and 55% relative humidity (rectangular markers).


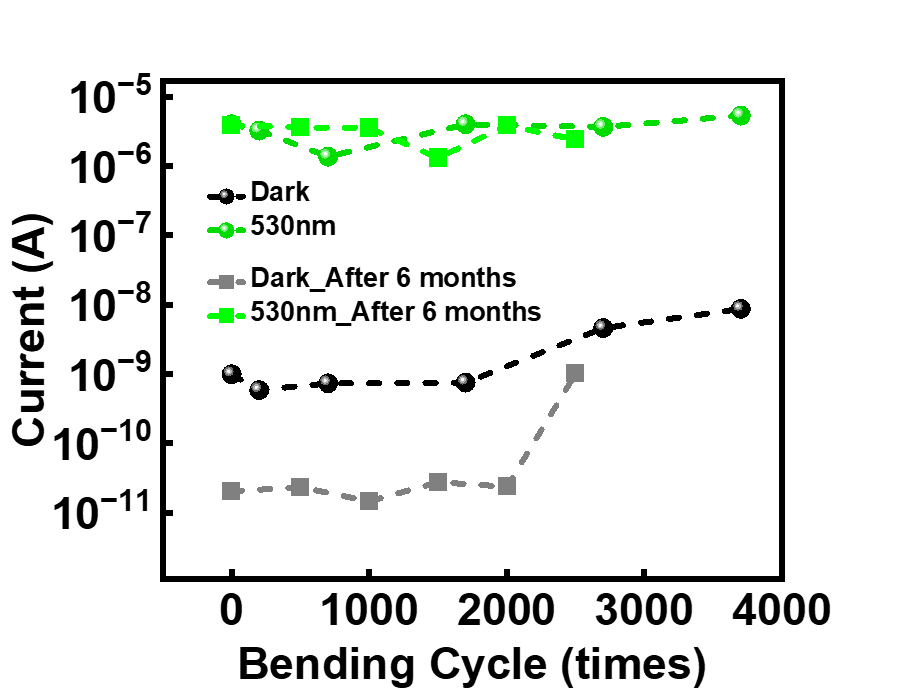


**Figure S10.** Current-voltage (I-V) characteristics of the photodiode (*f*_8_ ratio) measured in pristine condition and after exposure to thermal environments at (a) 60 °C and (b) 80 °C for 1 hour and 8 hours, respectively.

**
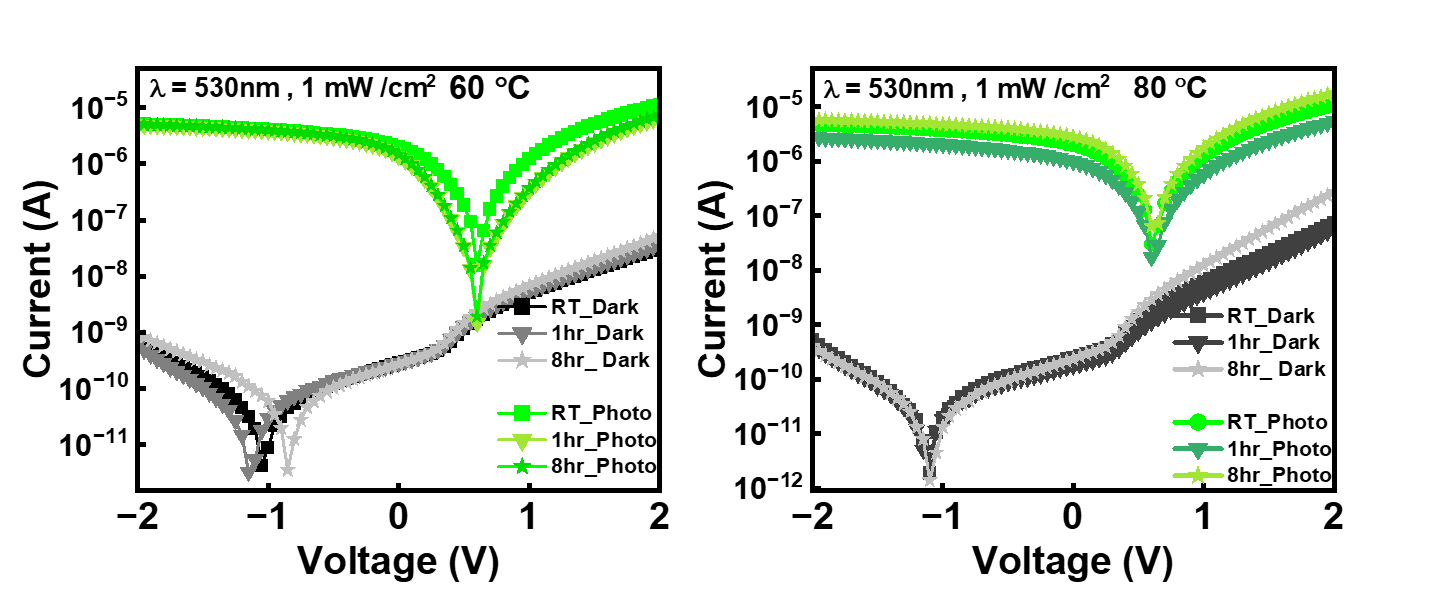
**

**Figure S11.** Time-dependent photocurrent characteristics of the flexible photodetector measured under continuous illumination for 3326 sec with the incident light power of 1 mW/cm^2^ and 30 mW/cm^2^.

**
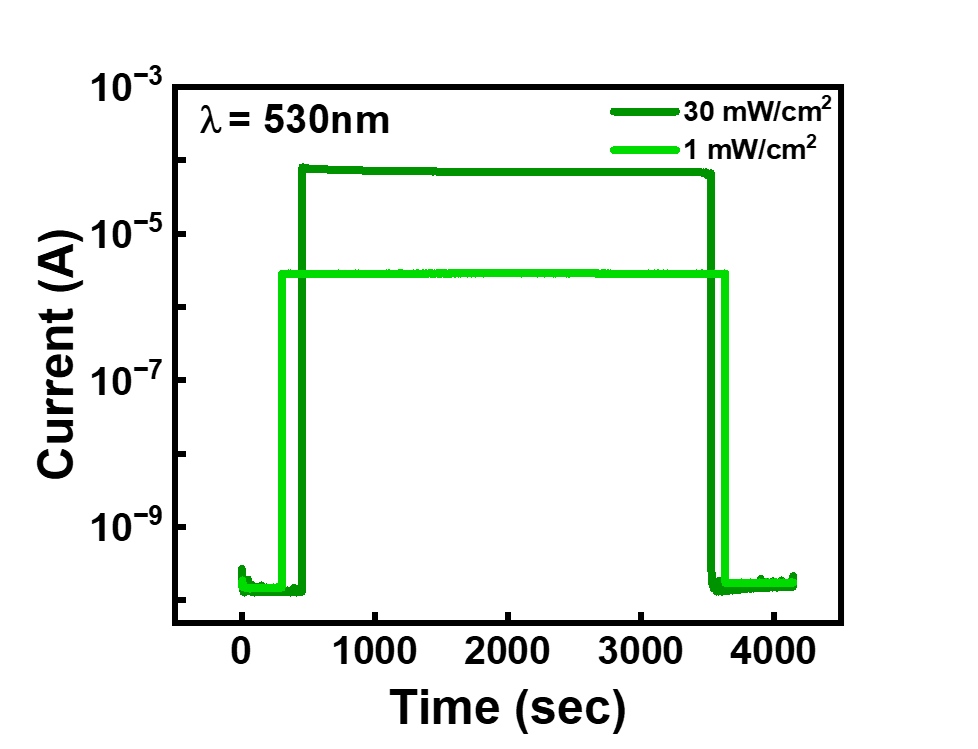
**

**Figure S12.** Comparison of responsivity of photodetectors in rigid or flexible devices with reported literature [1-17].


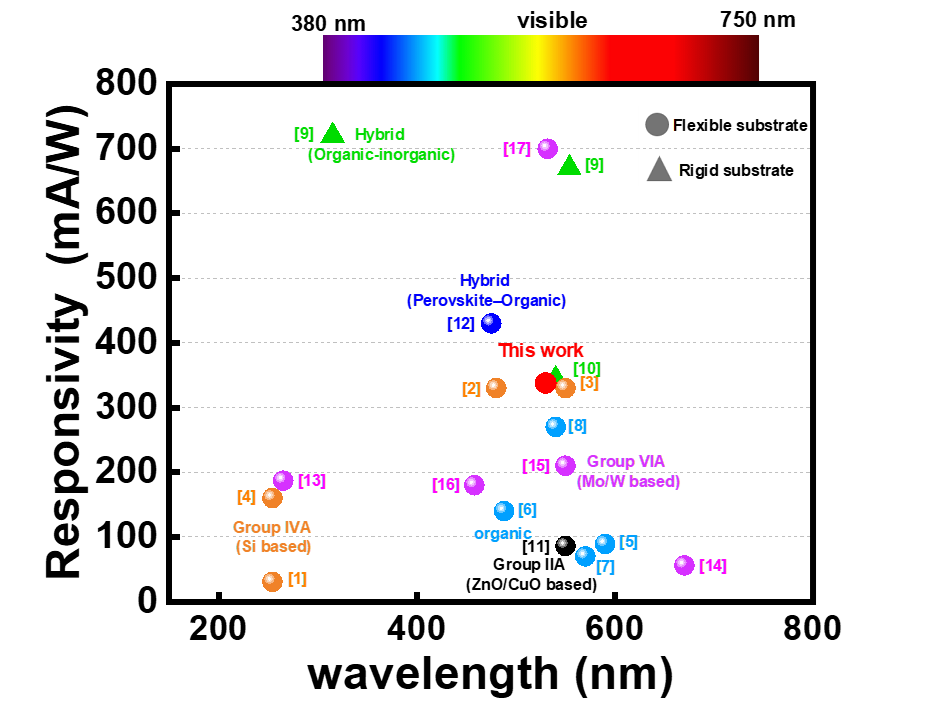


**Table S1.** Parameters used in AFORS-HET simulation for a-Si:H based VPDs.

| **Parameter** | **p-a-SiCx:H** | **i-a-Si:H, *f*_2_** | **i-a-Si:H, *f*_4_** | **i-a-Si:H, *f*_8_** | **i-a-Si:H, *f*_12_** |
| --- | --- | --- | --- | --- | --- |
| Layer thickness (nm) | 15 | 400 | 400 | 400 | 400 |
| Dielectric constant | 11.9 | 11.9 | 11.9 | 11.9 | 11.9 |
| Electron affinity (eV) | 3.9 | 3.9 | 3.9 | 3.9 | 3.9 |
| Band gap (eV) | 2.08 | 1.77 | 1.77 | 1.85 | 1.83 |
| Optical band gap (eV) | 2.08 | 1.77 | 1.77 | 1.85 | 1.83 |
| Valence band density (cm^-3^) | 1ⅹ10^20^ | 1ⅹ10^20^ | 1ⅹ10^20^ | 1ⅹ10^20^ | 1ⅹ10^20^ |
| Conduction band density (cm^-3^) | 1ⅹ10^20^ | 1ⅹ10^20^ | 1ⅹ10^20^ | 1ⅹ10^20^ | 1ⅹ10^20^ |
| Hole mobility (cm2V-1s-1) | 0.01 | 0.01 | 0.01 | 0.01 | 0.01 |
| Electron mobility (cm2V-1s-1) | 1 | 1 | 1 | 1 | 1 |
| Thermal velocity of electron (cm/s) | 1ⅹ10^7^ | 1ⅹ10^7^ | 1ⅹ10^7^ | 1ⅹ10^7^ | 1ⅹ10^7^ |
| Thermal velocity of hole (cm/s) | 1ⅹ10^7^ | 1ⅹ10^7^ | 1ⅹ10^7^ | 1ⅹ10^7^ | 1ⅹ10^7^ |
| Doping concentration (cm^-3^)  (activation energy used in i-a-Si:H) | 5.3ⅹ10^19^ | 0.61 eV | 0.72 eV | 0.89 eV | 0.74 eV |
| Layer density (g/cm^3^) | 2.328 | 2.328 | 2.328 | 2.328 | 2.328 |

**Reference**

[1] C. Hong, Y. Tao, V. K. Dat, J.-H. Kim, npj Flexible Electronics 2025, 9 (1), 13.

[2] D. S. Schneider, A. Bablich, M. C. Lemme, Nanoscale 2017, 9 (25), 8573.

[3] M. R. Esmaeili-Rad, S. Salahuddin, Sci Rep 2013, 3 (1), 2345.

[4] T. N. Ng, W. S. Wong, M. L. Chabinyc, S. Sambandan, R. A. Street, Applied Physics Letters 2008, 92 (21).

[5] N. Pandit, A. Mandal, R. Mandal, B. Mukherjee, Sensors and Actuators A: Physical 2024, 378, 115799.

[6] B. K. S. Reddy, S. Veeralingam, P. H. Borse, S. Badhulika, Materials Chemistry Frontiers 2022, 6 (3), 341.

[7] K. Y. Kim, S. H. Yoon, I. K. Kim, H. G. Kim, D.-k. Kim, E. Le Shim, Y. J. Choi, Nanotechnology 2019, 30 (43), 435203.

[8] H. Zhou, P. Gui, Q. Yu, J. Mei, H. Wang, G. Fang, Journal of Materials Chemistry C 2015, 3 (5), 990.

[9] G. de Cesare, F. Galluzzi, F. Irrera, D. Lauta, F. Ferrazza, M. Tucci, Journal of non-crystalline solids 1996, 198, 1189.

[10] D. Kannichankandy, P. M. Pataniya, C. K. Zankat, M. Tannarana, V. M. Pathak, G. K. Solanki, K. D. Patel, Applied Surface Science 2020, 524, 146589.

[11] T. Chen, Y. Fang, M. Zhu, Z. Zhao, W. Lei, Z. Zhu, H. Jiang, Sensors and Actuators A: Physical 2024, 365, 114820.

[12] K. Pu, Z. Xu, Y. Gao, C. Zhao, F. Ershad, Y. Li, R. Wang, C. Yu, G. Wei, Advanced Materials Technologies 2023, 8 (16), 2300207.

[13] D. B. Velusamy, M. A. Haque, M. R. Parida, F. Zhang, T. Wu, O. F. Mohammed, H. N. Alshareef, Adv. Funct. Mater. 2017, 27 (15), 1605554.

[14] G. Wu, R. Fu, J. Chen, W. Yang, J. Ren, X. Guo, Z. Ni, X. Pi, C. Z. Li, H. Li, Small 2018, 14 (39), 1802349.

[15] L. Cong, H. Zhou, M. Chen, H. Wang, H. Chen, J. Ma, S. Yan, B. Li, H. Xu, Y. Liu, Journal of Materials Chemistry C 2021, 9 (8), 2806.

[16] R. Zhuo, D. Wu, Y. Wang, E. Wu, C. Jia, Z. Shi, T. Xu, Y. Tian, X. Li, Journal of Materials Chemistry C 2018, 6 (41), 10982.

[17] C. Li, H. Wang, F. Wang, T. Li, M. Xu, H. Wang, Z. Wang, X. Zhan, W. Hu, L. Shen, Light: Sci. Appl. 2020, 9 (1), 31.
